# *Bacillus coagulans* TL3 Inhibits LPS-Induced Caecum Damage in Rat by Regulating the TLR4/MyD88/NF-*κ*B and Nrf2 Signal Pathways and Modulating Intestinal Microflora

**DOI:** 10.1155/2022/5463290

**Published:** 2022-02-07

**Authors:** Yan Wang, Jiaxi Lin, Ziyang Cheng, Tiancheng Wang, Jia Chen, Miao Long

**Affiliations:** College of Animal Science & Veterinary Medicine, Shenyang Agricultural University, Shenyang 110866, China

## Abstract

**Background:**

*Bacillus coagulans* has been widely used in food and feed additives, which can effectively inhibit the growth of harmful bacteria, improve intestinal microecological environment, promote intestinal development, and enhance intestinal function, but its probiotic mechanism is not completely clear.

**Aim:**

The aim of this study is to discuss the effect and mechanism of *Bacillus coagulans* TL3 on oxidative stress and inflammatory injury of cecum induced by LPS.

**Method:**

The Wistar rats were randomly divided into four groups, each containing 7 animals. Two groups were fed with basic diet (the LPS and control, or CON, groups). The remaining groups were fed with basic diet and either a intragastric administration high or low dose of *B. coagulans*, forming the HBC and LBC groups, respectively. The rats were fed normally for two weeks. On the 15th day, those in the LPS, HBC, and LBC groups were injected intraperitoneally with LPS—the rats in the CON group were injected intraperitoneally with physiological saline. After 4 hours, all the rats were anesthetized and sacrificed by cervical dislocation, allowing samples to be collected and labeled. The inflammatory and antioxidant cytokine changes of the cecum were measured, and the pathological changes of the cecum were observed, determining the cecal antioxidant, inflammation, and changes in tight junction proteins and analysis of intestinal flora.

**Result:**

The results show that LPS induces oxidative damage in the cecal tissues of rats and the occurrence of inflammation could also be detected in the serum. The Western blot results detected changes in the NF-*κ*B- and Nrf2-related signaling pathways and TJ-related protein levels. Compared with the LPS group, the HBC group showed significantly downregulated levels of expression of Nrf2, NQO1, HO-1, GPX, and GCLC. The expression of TLR4, MYD88, NF-*κ*B, IL-6, TNF*α*, and IL-1*β* was also significantly downregulated, while the expression of other proteins (ZO-1, occludin, and claudin-1) increased significantly. *Bacillus coagulans* TL3 was also found to increase the relative abundance of the beneficial bacterium *Akkermansia muciniphila* in the intestines. There is also a significant reduction in the number of harmful bacteria *Escherichia coli* and Shigella (Enterobacteriaceae).

**Conclusion:**

*Bacillus coagulans TL3* regulates the TLR4/MyD88/NF-*κ*B and Nrf2 signaling pathways in the cecal tissue of rats, protects the intestine from inflammation and oxidative damage caused by LPS, and inhibits the reproduction of harmful bacteria and promotes beneficial effects by regulating the intestinal flora bacteria grow, thereby enhancing intestinal immunity.

## 1. Introduction

The intestine is an important organ for digesting food and feed, but it is often damaged by various intestinal diseases. Intestinal barrier refers to the structure and function of the intestinal tract that prevents harmful substances such as bacteria and toxins from passing through the intestinal mucosa into other tissues, organs, and blood circulation in the human body. It includes intestinal mucosal epithelium, intestinal mucus, intestinal flora, secretory immunoglobulin, and intestinal-related lymphoid tissue [1]. Therefore, the destruction of the intestinal barrier will lead to increased intestinal permeability, and pathogens enter the body and trigger systemic inflammation, which is the main pathogenesis of most intestinal diseases [2]. Oxidative stress is believed to play a key role in the development of intestinal injury in inflammatory bowel disease [3–5]. Oxidative stress refers to the increase in the level of oxygen free radicals in cells, which leads to the damage of lipids, proteins, and DNA. It is related to many pathological conditions, as well as the increase of reactive oxygen species (ROS) and lipid peroxidation [6, 7]. The production of high levels of free radicals in the intestine can produce cytotoxic effects on intestinal epithelial cell membrane phospholipids [8].

Studies have shown that LPSs are the main components of the outer membranes of Gram-negative bacteria. They can be used as an effective activator of innate immune response, leading to the production of pro- and anti-inflammatory mediators. As a result, LPSs are widely used to induce immune inflammation [9–11]. Other studies have shown that LPSs can induce the formation of ROS intermediaries which accounts for some of their toxicity [12]. Excessive ROS production (or poor ability to eliminate active in-termediaries) leads to a continuous imbalance in redox homeostasis, resulting in endogenous oxidative stress [13, 14]. Oxidative stress can cause DNA hydroxylation, protein denaturation, lipid peroxidation, and intestinal damage [15]. Therefore, LPSs have long been used to induce oxidative stress in experimental animals [12]. The intestine is the main part of the body in which nutrient absorption takes place and is also one of the organs targeted by LPSs [16]. LPSs can directly trigger inflammatory responses in intestinal epithelial cells and destroy the integrity of the intestinal mucosal barrier [17]. As a result, LPSs are widely used to establish intestinal mucosal barrier injury models [18, 19].

Probiotics are a potential strategy to prevent and treat many gastrointestinal diseases including viral diarrhea, antibiotic-associated diarrhea, bacterial infections, and inflammatory bowel disease [20]. Probiotics can protect the health of animals by maintaining the balance of intestinal microbes. They have various intestinal protective effects including increasing the mucus layer and strengthening tight junctions (TJs) to protect the integrity of the intestinal epithelium [21, 22]. Little is known about the beneficial mechanism of probiotics, but it is likely to be immunomodulatory and anti-inflammatory, such as downregulating the expression of inflammatory cytokines and Toll-like receptors [23, 24]. Related studies have shown that probiotics change the mucosal immune system through the process of Toll-like receptors, inhibit the NF-*κ*B signaling pathway, upregulate anti-inflammatory cytokines, such as IL-10, and reduce proinflammatory cytokines, such as TNF-*α* [25–28]. In addition, probiotics can improve the barrier function by promoting the synthesis of tightly connected proteins [29]. Probiotics inhibit the growth of potential pathogenic bacteria by producing bacteriocins and create a more acidic environment that is harmful to inflammatory bacteria but promotes the growth of beneficial species, such as Lactobacillus and Bifidobacterium, thereby beneficially regulating the composition of the microflora [30, 31].

Among a variety of probiotic strains, *Bacillus coagulans* has a variety of probiotic effects, and others have also proved its safety by feeding it to mice [32]. *B. coagulans* can promote the digestion and absorption of nutrients. As a probiotic, *Bacillus coagulans* has the advantage that the spores formed have strong resistance to extreme environmental conditions. In addition, compared with other probiotics, Bacillus has higher acid resistance and better stability during heat treatment and low-temperature storage [33, 34]. In addition, the spores of *Bacillus coagulans* have strong resistance, reactivity, and stability. They can be activated in the acidic environment of the stomach and begin to germinate and proliferate in the intestines [35]. The spores can adapt to the low-oxygen environment of the intestinal tract and reach the gastrointestinal tract smoothly, thereby playing the role of lactic acid bacteria in the intestinal tract. *B. coagulans* does so by promoting the secretion of digestive enzymes, thus producing a variety of metabolites by itself and by degrading polymer compounds. It also improves body's digestive function by improving the vitality of intestinal digestive enzymes [36, 37]. It has also been shown that *B. coagulans* can degrade macromolecular substances via metabolic pathways in the body [38]. Reports indicate that certain active ingredients in the fermentation supernatant of *B. coagulans* can form a biological protective barrier in the intestinal tract of the body, promoting the immune response of the digestive tract mucosa, and thereby improving intestinal immunity [39]. In addition, *Bacillus coagulans* can produce a bacteriocin called coagulation protein, which has activity against a broad spectrum of intestinal microorganisms [35]. Related research shows that *Bacillus coagulans* can reduce the rate of diarrhea and improve the growth performance of piglets [40]. At the same time, *Bacillus coagulans* can significantly increase the final weight, daily gain, and relative weight gain of shrimp [35]. *Bacillus coagulans* is expected to be used as a probiotic feed additive into the breeding industry. However, the mechanism of the strains to protect intestinal tract is not fully clear.

The purpose of this study is to study the effect and mechanism of *Bacillus coagulans* TL3 on oxidative stress and inflammatory injury of cecum induced by lipopolysaccharide (LPS) and to discuss whether probiotics can affect the immune function of intestinal tract, so that the intestinal tract can directly resist the intestinal injury induced by LPS, which lays a theoretical foundation for the toxicological study and clinical application of *Bacillus coagulans*.

## 2. Materials and Methods

### 2.1. Chemicals, Strains, and Media

The LPS (from *Escherichia coli* O55:B5) and MRS (De Man Rogosa Sharpe) broth used were purchased from Solarbio (Beijing, China). The *B. coagulans* TL3 strain was isolated and preserved by the laboratory of the College of Animal Husbandry and Veterinary Medicine, Shenyang Agricultural University. *B. coagulans* TL3 forms milky-white colonies of uniform size with smooth surfaces and neat edges. It is a rod-shaped bacterium according to Gram stain imaging and microscopic examination. Furthermore, it is a facultative anaerobic Gram-positive *bacillus*. The 16S rRNA sequencing results for this strain (GenBank number: MK685114.1) show that 99% of its DNA sequence is homologous to the DNA of *B. coagulans* listed by the NCBI. Through its physiological and biochemical characteristics (and molecular biology identification procedures), the strain was further identified as *B. coagulans*.

### 2.2. Animal and Experimental Design

*B. coagulans* TL3 was prepared by night culture and plate counting and then diluted to the corresponding colony number with medium. Male Wistar rats (6 weeks old, weight 180 ± 10 g) were purchased from China Liaoning Changsheng Biotechnology Co., Ltd. The rats were maintained in SPF conditions and entry restricted. The humidity was maintained at 45–55%, and the rats subjected to light–dark cycles lasting 12 hours each. The temperature was kept at 23 ± 2°C.The rats were given an adaptation period of 1 week before the start of the experiments. The experiments were carried out according to the care principles specified for SPF laboratory animals. The experiments were permitted by the Shenyang Agricultural University under the Laboratory Animal Care Ethics Committee (People's Republic of China Animal Ethics Regulations and Guidelines) for animal experiments ((Permit No. 264SYXK<Liao>2011-0001, September 2018).

**Figure 1:**
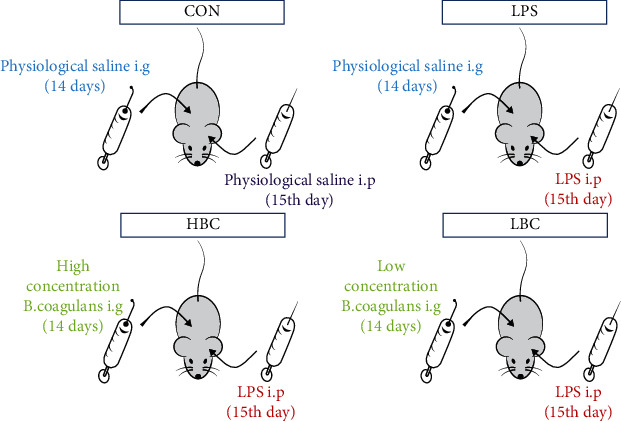
Animal and experimental design.

The animal testing were carried out according to the U.K. Animals (Scientific Procedures) Act, 1986 and the relevant guidelines, Directive 2010/63/EU. The Wistar rats were randomly divided into four groups, each containing 7 animals. Two groups were fed with basic diet (the LPS and control, or CON, groups). The remaining groups were fed with basic diet and either a intragastric administration high (1 mL of 1.2 × 10^8^ CFU/mL TL3) or low (1 mL of 1.2 × 10^3^ CFU/mL TL3) dose of *B. coagulans*, forming the HBC and LBC groups, respectively. The rats were fed normally for two weeks. On the 15th day, those in the LPS, HBC, and LBC groups were injected intraperitoneally with LPS (1 mg/kg body weight)—the rats in the CON group were injected intraperitoneally with physiological saline (Figure 1). After 4 hours [41], all rats were anesthetized and sacrificed by cervical dislocation. Samples of cecal tissue and contents were collected and labeled. The blood was collected by enucleating the eyeball. All samples are stored in a refrigerator at -80 degrees for subsequent testing.

### 2.3. Enzyme-Linked Immunosorbent Assay (ELISA)

After standing at room temperature for 30 minutes, serum was obtained from the blood and centrifuged at 3000 g for 10 minutes at 4°C. ELISA kits (Jiangsu Enzyme Industry Co., Ltd., Jiangsu, China) were then used according to manufacturer's protocols to analyze the levels of IL-1*β* (interleukin 1*β*), IL-6 (interleukin-6), IL-4 (interleukin 4), and TNF-*α* (tumor necrosis factor alpha) in the serum. The absorbances of each sample and appropriate standards were then measured at a wavelength of 450 nm. The detection range of IL-1 *β* ELISA kit is 1 ng/L-40 ng/L. The detection range of IL-4 and IL-6 ELISA kit is 3 pg/mL-120 pg/mL. The detection range of TNF-*α* ELISA kit is 10 ng/L-360 ng/L. The correlation coefficient *R* between the linear regression of the sample and the expected concentration is more than 0.95. The intrabatch coefficient of variation and interbatch coefficient of variation should be less than 10% and 12%, respectively.

### 2.4. Antioxidative Stress Indicators

Take 0.020-1 g fresh tissue, rinse with PBS at 2-8°C (0.01 M pH 7.4), remove blood, dry with filter paper, weigh it, put it into a homogenizing container, add 2-8°C PBS, to homogenate according to the ratio of weight (g): volume (mL) = 1 : 9, centrifuge at 4°C, 10000 × g for 10 min, take the supernatant, and put it on the ice to be tested. The levels of malondialdehyde (MDA), catalase (CAT), glutathione peroxidase (GSH-Px), and superoxide dismutase (SOD) in the rat tissues were measured using commercially available reagents by colorimetry (Elite Biotechnology Co., Ltd., Wuhan, China). A Sunrise microplate reader (Tecan, Mannedorf, Switzerland) was used to measure the optical densities of the samples and thus determine their concentrations.

### 2.5. Histological Evaluation

Take each group of rat cecum tissue specimens fixed in advance with tissue fixative, mark them, make pathological sections, stain them with hematoxylin and eosin (HE), and finally, observe the lesions with a microscope. This process is produced by Wuhan Saiweier Company (Sevier Biological technology Co. Ltd., Wuhan, China).

### 2.6. Western Blot Analysis

Mammalian total protein extraction kits (ProteinExt, TransGen Biotech, Beijing, China) were used to obtain total protein from the cecal tissues. Protein quantification kits (Easy II, TransGen Biotech, Beijing, China) were used to determine protein concentrations. The proteins were separated using sodium dodecyl sulphate polyacrylamide gel electrophoresis and transferred to PVDF membranes (Solarbio, Beijing, China). The membranes were incubated overnight at 4°C with 5% BSA and then incubated with primary antibodies (Nrf2, NQO1, HO-1, GPX, GCLC, TLR4, MYD88, NF-*κ*B, IL-1*β*, IL-6, TNF-*α*, ZO-1, occludin, and claudin-1) below 4°C. The membranes were then washed with TBST and incubated with secondary antibody blocking solution for 2 hours at room temperature. A bioimaging system (DNR Bio-Imaging Systems) was used to detect proteins using an NCM ECL ultra-assay kit used according to manufacturer's instructions (NCM Biotech, Suzhou, China). The expression levels of the target proteins were quantified using the gel quantification system.

### 2.7. Analysis of Intestinal Flora

The contents of rat's cecum were collected in a sterile cryopreservation tube, and 16S rRNA gene amplification products were generated using Illumina sequencing technology to determine the diversity of bacterial species in the rat intestinal flora and perform structural comparison and function prediction. In this process, the sample processing and analysis are carried out by Beijing Biomec (Beijing, BioMark Biotechnology Co., Ltd.).

### 2.8. Statistical Analysis

Each experiment was repeated at least three times. The experimental data were initially analyzed using Microsoft Excel and then tested for significance using one-way ANOVA tests (using the SPSS software package v17.0). All numerical results are expressed in the form mean ± standard error and *p* < 0.05 represents a statistically significant difference between test groups. All the graphs presented in this work were constructed and drawn using GraphPad Prism v5 and Microsoft Office software.

## 3. Results

### 3.1. H&E Staining Results

**Figure 2:**
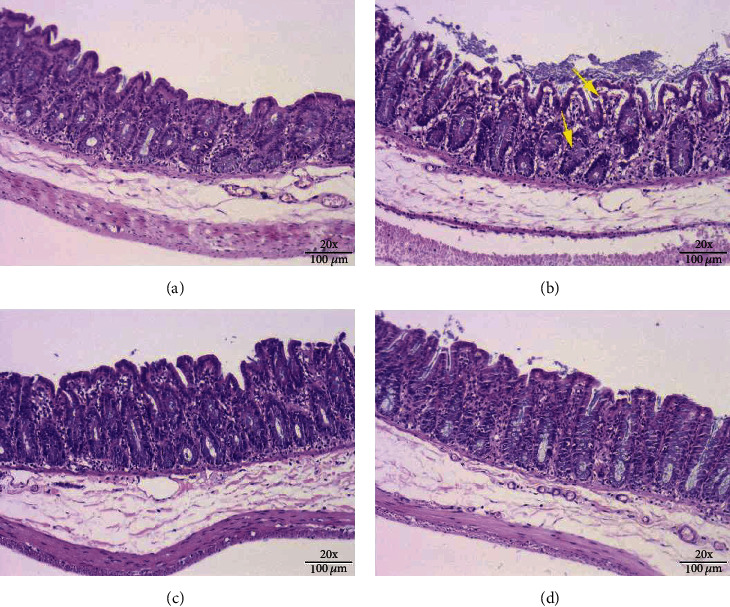
H&E stained images showing the structures of the ceca of rats from the different experimental groups: (a) control group, (b) LPS group, (c) HBC group, and (d) LBC group (×200 magnification). The yellow arrow indicates the pathological damage in the cecum, such as the destruction or rupture of the intestinal gland structure.

Figure 2 shows images of rat cecum tissues obtained via H&E staining. In the image obtained from a rat in the control group (Figure 2(a)), the structure of the cecum intestinal gland can be clearly seen neatly arranged in the lamina propria without breakage. In contrast, in the image obtained from the rat selected from the LPS group (Figure 2(b)), the structure of the intestinal glands is disordered, and there are many ruptures. There is also a certain degree of edema, and the thickness of the muscularis mucosa is inconsistent but significantly thinner. Compared with the images in Figure 2(b) (LPS group), the images in Figure 2(c) (HBC group) and Figure 2(d) (LBC group) showed a significant improvement in the structure of the cecum with almost no abnormalities, which are very close to the images of the control group rats..

### 3.2. Alcian Blue Staining Results

**Figure 3:**
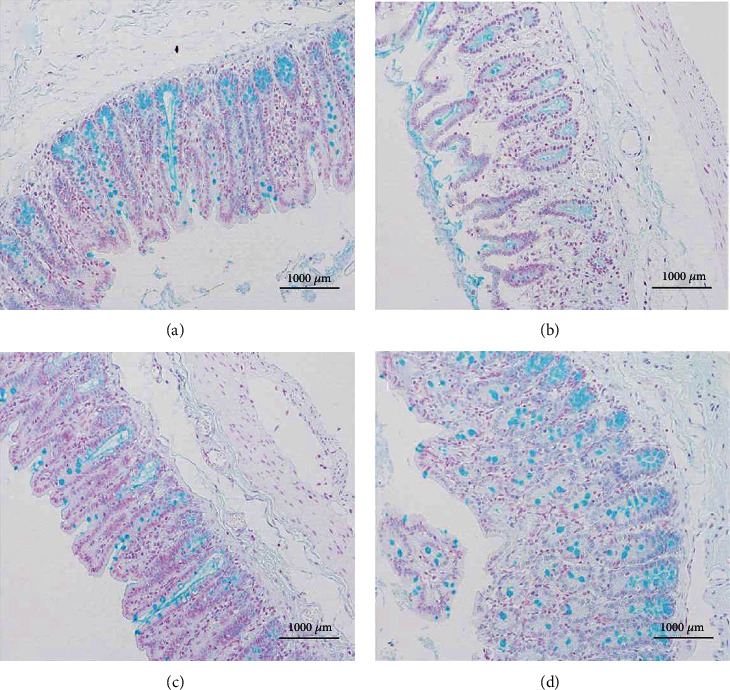
Alcian blue stained images showing the structures of the ceca of rats from the different experimental groups: (a) control group, (b) LPS group, (c) HBC group, and (d) LBC group (×200 magnification).

Figure 3 shows sections of rat ceca stained using alcian blue. It is clear that the number of goblet cells in the image obtained using a rat from the LPS group (Figure 3(b)) is significantly reduced compared with the image obtained using a rat from the control group (Figure 3(a)). The structure of the cecal tissue is also rather disordered.

Compared with Figure 3(b), the corresponding images obtained for the HBC group (Figure 3(c)) and LBC group (Figure 3(d)) have significantly improved numbers of goblet cells in the cecum, and the structures of the ceca are restored to that of normal tissue, having appearances very close to that of the control group. The results showed that TL3 can stabilize the number of goblet cells in the cecum of rats, maintain the intestinal barrier, and prevent damage caused by LPS.

### 3.3. IL-6, IL-4, IL-1*β*, and TNF-*α* Concentrations in the Rat Sera

**Figure 4:**
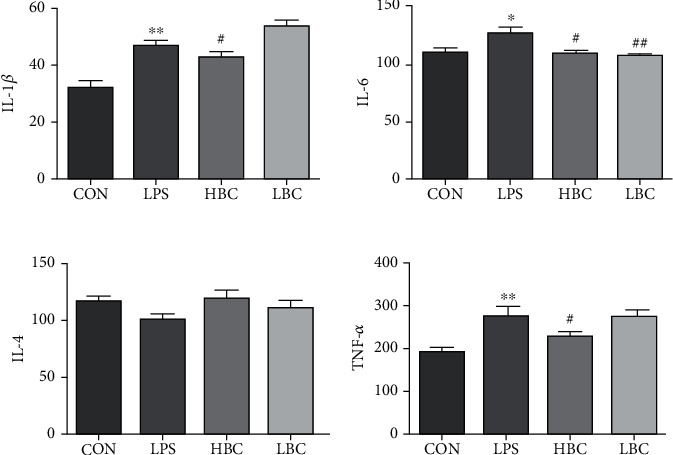
Detection results obtained for the levels of IL-1*β*, IL-6, IL-4, and TNF-*α* in the sera of rats in the four test groups: CON (blank control group), LPS (LPS toxin group), HBC (high concentration *B. coagulans* + LPS group), and LBC (low concentration *B. coagulans* + LPS group). The symbol “^∗^” indicates a significantly different result compared to the control group (*p* < 0.05) and “^∗∗^” a highly significant difference (*p* < 0.01). Similarly, “^#^” represents a significantly different result compared to the LPS group (*p* < 0.05) and “^##^” a highly significant difference (*p* < 0.01).

The test results are shown in Figure 4. They indicate that the addition of LPS significantly increases the levels of IL-1*β*, TNF-*α*, and IL-6 in the rat serum samples compared to the control group. At the same time, the IL-4 level is reduced. Compared with the LPS group, the levels of IL-1*β*, TNF-*α*, and IL-6 in the sera of the rats in the HBC group were significantly reduced (*p* < 0.05), and the IL-4 level was increased. Overall, the HBC group showed the smallest differences in serum parameters compared to the control group. The IL-*β* of the LBC group increased compared with the LPS group, and the IL-6 and TNF-*α* of LBC both decreased. Among them, IL-6 was extremely significant. Compared with the LPS group, IL-4 had elevated but not significant. It shows that the protective effect of low-dose *Bacillus coagulans* on the intestinal tract is slightly worse. The results indicate that the addition of *B. coagulans* to the feed can alleviate or prevent the toxic effects of LPS in rats.

### 3.4. Oxidative and Antioxidant Markers in Rat Cecal Tissues

**Figure 5:**
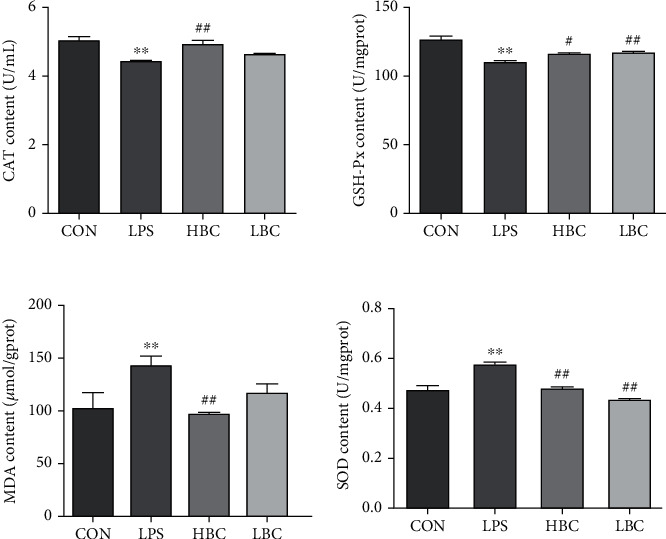
MDA: malondialdehyde; CAT, catalase; SOD: superoxide dismutase; GSH-Px: glutathione peroxidase; levels measured in the cecal tissues of the rats (CON: blank control group; LPS: LPS toxin group; HBC: high dose of *B. coagulans* + LPS group; LBC: low dose of *B. coagulans* + LPS group). The symbol “^∗^” indicates a significantly different result compared to the control group (*p* < 0.05) and “^∗∗^” a highly significant difference (*p* < 0.01). Similarly, “^#^” represents a significantly different result compared to the LPS group (*p* < 0.05) and “^##^” a highly significant difference (*p* < 0.01).

The oxidation indicators CAT, MDA, GSH-Px, and SOD were measured using oxidation kits giving the results shown in Figure 5. As can be seen, the CAT and GSH-Px activities were extremely significantly reduced (*p* < 0.01) in the LPS group compared with the control group. On the other hand, the MDA content and SOD activity were extremely significantly increased (*p* < 0.01).

The addition of the probiotic to the HBC group not only reverses the increase in MDA content and SOD activity induced by LPS (*p* < 0.01) but also significantly increases the CAT and GSH-Px activities (CAT: *p* < 0.01, GSH-Px: *p* < 0.05) compared with the LPS group. Moreover, there was also significantly reduced SOD activity (*p* < 0.01) and increased GSH-Px activity (*p* < 0.01) in the LBC group compared with the LPS group.

These results indicate that the addition of *B. coagulans* can effectively protect the ceca of rats from LPS-induced oxidative damage.

### 3.5. Expression of Proteins Related to Antioxidant Damage

**Figure 6:**
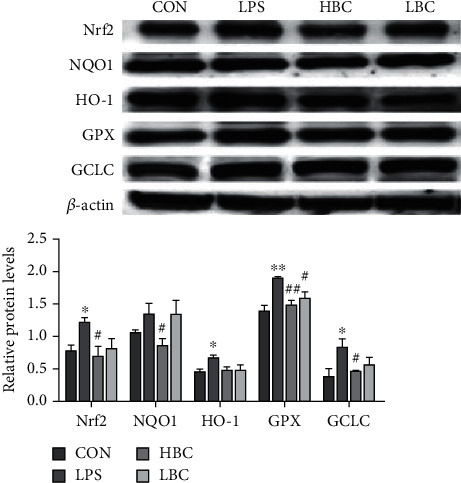
The changes in Nrf2, NQO1, HO-1, GPX, and GCLC protein expression levels in the cecum tissues of rats in the different experimental groups (CON: blank control group; LPS: LPS toxin group; HBC: high dose of *B. coagulans* + LPS group; LBC: low dose of *B. coagulans* + LPS group).The symbol “^∗^” indicates a significantly different result compared to the control group (*p* < 0.05) and “^∗∗^” a highly significant difference (*p* < 0.01). Similarly, “^#^” represents a significantly different result compared to the LPS group (*p* < 0.05) and “^##^” a highly significant difference (*p* < 0.01).

The measured levels of proteins related to antioxidative damage are shown in Figure 6 for the different groups. The results show that the expression of Nrf2, NQO1, HO-1, GPX, and GCLC is increased in the cecum tissues of the rats in the LPS group compared with those in the control group (GPX, *p* < 0.01; Nrf2, HO-1, GCLC, *p* < 0.05). Furthermore, compared with the LPS group, the expression of GPX, Nrf2, HO-1, and GCLC is significantly reduced in the HBC group (GPX, *p* < 0.01; Nrf2, HO-1, GCLC, *p* < 0.05).

There were no significant differences between the results obtained for the LBC and LPS groups except that the GPX level was significantly reduced (*p* < 0.05). Thus, the addition of a low dose (1.2 × 10^3^ CFU/mL) of the probiotic provides little protection against the oxidative damage caused by LPS. In contrast, a high dose (1.2 × 10^8^ CFU/mL) provides a strong protective effect.

### 3.6. Expression of Proteins Related to Inflammation

**Figure 7:**
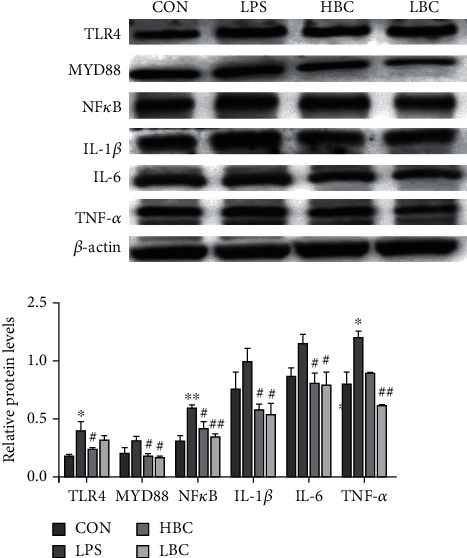
The change in TLR4, MYD88, NF-*κ*B, IL-1*β*, IL-6, and TNF-*α* protein expression levels in the cecum tissues of rats in the different experimental groups. The symbol “^∗^” indicates a significantly different result compared to the control group (*p* < 0.05) and “^∗∗^” a highly significant difference (*p* < 0.01). Similarly, “^#^” represents a significantly different result compared to the LPS group (*p* < 0.05) and “^##^” a highly significant difference (*p* < 0.01).

As can be seen from Figure 7, the expression levels of the proteins TLR4, MYD88, NF-*κ*B, IL-1*β*, IL-6, and TNF-*α* are all increased in the cecum tissues of the rats in the LPS group compared to those in the control group (NF-*κ*B, *p* < 0.01; TLR4, TNF-*α*, *p* < 0.05). In contrast, in addition to TNF-*α*, there were greatly reduced levels of TLR4, MYD88, NF-*κ*B, IL-1*β*, and IL-6 in the HBC group (*p* < 0.05).

It is worth noting that the LBC group also exhibits significantly reduced levels of expression of TNF-*α*, NF-*κ*B, MYD88, IL-1*β*, and IL-6 (TNF-*α*, NF-*κ*B, *p* < 0.01; MYD88, IL-1*β*, IL-6, *p* < 0.05). The results therefore show that the rats in both the HBC and LBC groups experienced a protective effect against the inflammatory damage caused by LPS.

### 3.7. Expression of TJ-Related Proteins

**Figure 8:**
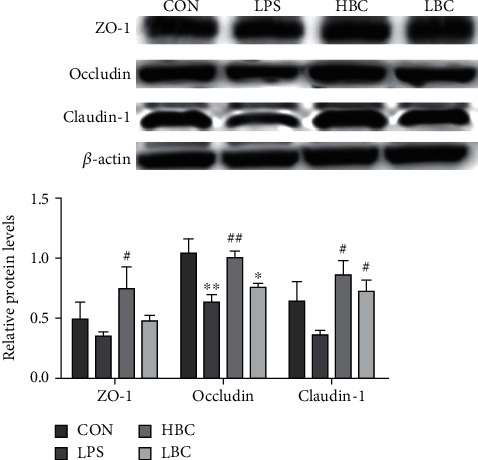
The change in ZO-1, occludin, and claudin-1 protein expression levels in the cecum tissues of rats in the different experimental groups (CON: blank control group; LPS: LPS toxin group; HBC: high dose of *B. coagulans* + LPS group; LBC: low dose of *B. coagulans* + LPS group). The symbol “^∗^” indicates a significantly different result compared to the control group (*p* < 0.05) and “^∗∗^” denotes a highly significant difference (*p* < 0.01). Similarly, “^#^” indicates a significantly different result compared to the LPS group (*p* < 0.05) and “^##^” a highly significant difference (*p* < 0.01).

As can be seen from Figure 8, the expression levels of the proteins ZO-1, occludin, and claudin-1 in the cecum tissues of the rats in the LPS group are reduced compared to those in the control group (occludin, *p* < 0.01). In contrast, the rats in the HBC group exhibit greatly increased levels of ZO-1, claudin-1, and occludin (ZO-1, claudin-1, *p* < 0.05; occludin, *p* < 0.01). The rats in the LBC group also showed a significantly increased level of expression of claudin-1 (*p* < 0.05).

The results show that a low dose of the probiotic (LBC group) reduces the damage caused by LPS, although the effect is not particularly obvious. On the other hand, a high dose (HBC group) can effectively protect the intestinal mucosal barrier and leave the intestinal tissue more complete and free from damage.

### 3.8. Effect of *B. coagulans* TL3 on the Intestinal Microbes Induced by LPS

To investigate the effect of TL3 on the change in intestinal flora in the rats induced by LPS, we evaluated the contents of the rata ceca by 16S rRNA sequencing. Contents of samples were obtained from all 28 rat specimens. A total of 143,151 circular consensus sequences (CCSs) were obtained after barcode identification, each sample producing at least 5,557 CCSs; the average number of CCSs was 5,965.

**Figure 9:**
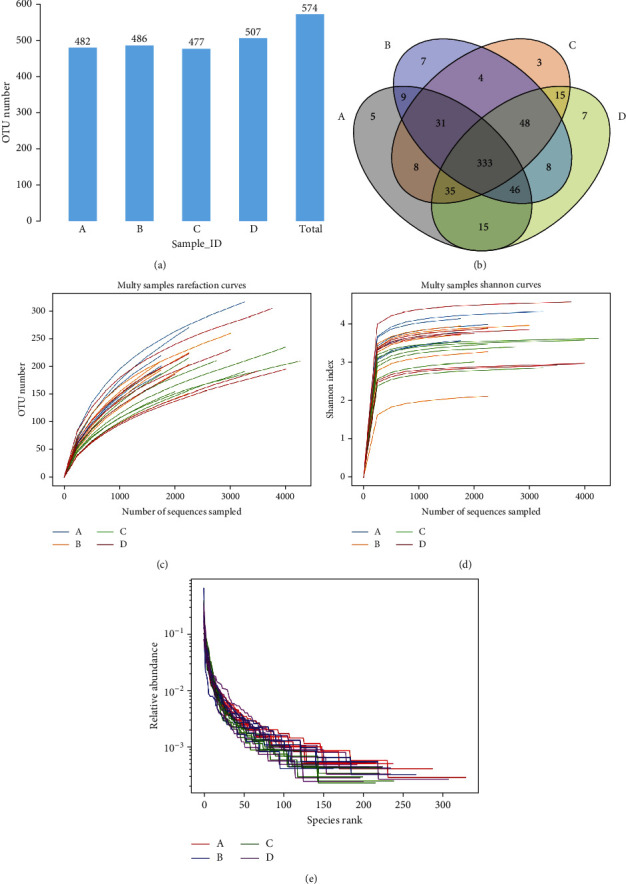
16S rRNA sequencing results showing (a) the number of OTUs, (b) a Venn diagram for the OTUs, (c) the rarefaction curve, (d) Shannon index curve, and (e) rank abundance curve.

A total of 574 operational taxonomic units (OTUs) were obtained by cluster analysis, as shown in Figure 9(a). The rats in the HBC group had a reduced number of OTUs compared with those in the LPS group but the difference was not significant. A Venn diagram was constructed using the results (Figure 9(b)). It clearly shows that there are 5, 7, 3, and 7 unique OTUs in the CON, LPS, HBC, and LBC groups, respectively.

The dilution, aroma index, and grade abundance curves (Figures 9(c)–9(e)) indicate that sufficient CCSs for 16S rRNA gene analysis had been obtained.

**Figure 10:**
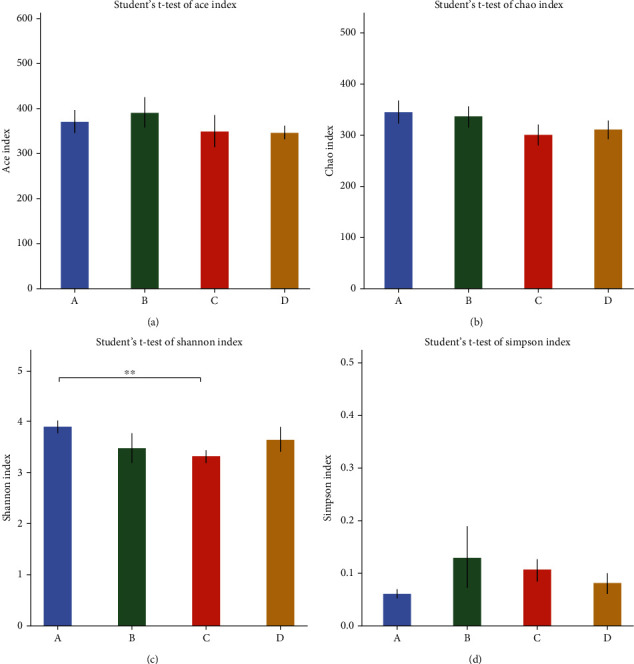
Various indices related to abundance and diversity of the microbiota present: (a) Ace index, (b) Chao index, (c) Shannon index, and (d) Simpson index. The symbol “^∗∗^” denotes a highly significant difference (*p* < 0.01).

The concept of alpha diversity is used to reflect the abundance and diversity of the species in a single sample. The Chao and Ace indices are used to measure species abundance, while the Simpson and Shannon indices measure species diversity. The values of these indices calculated for the different experimental groups are shown in Figure 10. As can be seen, the results obtained for the indices are consistent with the trends observed in the number of OTUs. The addition of TL3 reduces the abundance and diversity of the microbiota induced in the rat gut by LPS but none of the differences are significant.

**Figure 11:**
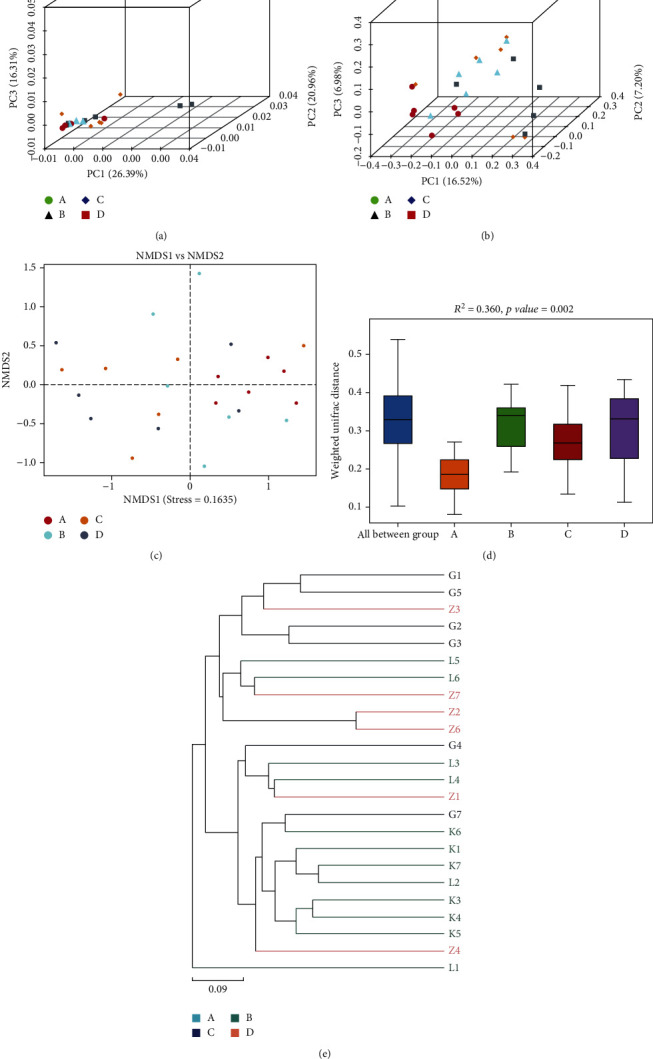
Multivariate analysis results showing (a) PCA, (b) PCoA, and (c) NMDS plots, (d) PERMANOVA results, and (e) UPGMA dendrogram.

Figure 11(a) illustrates a PCA analysis chart. Each colored dot represents a group. The more similar the species of each sample, the closer the distance between the two samples. From this figure, the degree of difference between individuals and groups can be observed. Among them, the contribution rates of the three principal components to the sample difference are 26.39%, 16.31%, and 20.96%, respectively.  Figure 11(b) shows PCoA analysis, which is an analysis based on a distance matrix calculated by species composition. Through principal coordinate analysis, multiple samples can be classified, and the diversity of samples can be revealed.  Figure 11(b) shows that the contribution rates of the three principal components to the sample difference are 6.98%, 16.32%, and 7.20%, respectively. The nonmetric multidimensional calibration method (NMDS) is a sorting method suitable for ecological research. It mainly reduces the research object (sample or variable) in the multidimensional space to a low-dimensional space for positioning, analysis, and classification, while retaining the object data analysis method of the original relationship between. The points in the figure represent each sample, and different colors represent different groups. The distance between points represents the degree of difference. The stress value of 0.1635 is less than 0.2, indicating that the NMDS analysis has a certain degree of reliability. The closer the sample is on the graph, the higher the similarity. *β* diversity analysis showed that there was significant difference between the HBC group and each group. Therefore, TL3 significantly changes the microbial composition of the cecum during inflammation caused by LPS, which may be one of the reasons why LPS reduces intestinal damage. The PER-MANOVA test results (Figure 11(d)) also showed significant differences (*R*^2^ = 0.365, *p* = 0.001). The weighted Bray-Curtis cluster tree diagram (Figure 11(e)) was made using the unweighted pair group method based on arithmetic average (UPGMA). The closer the samples, the shorter the branch length, indicating that the species composition of the two samples are more similar, revealing the regulatory effect of TL3 on the structure of intestinal microflora.

**Figure 12:**
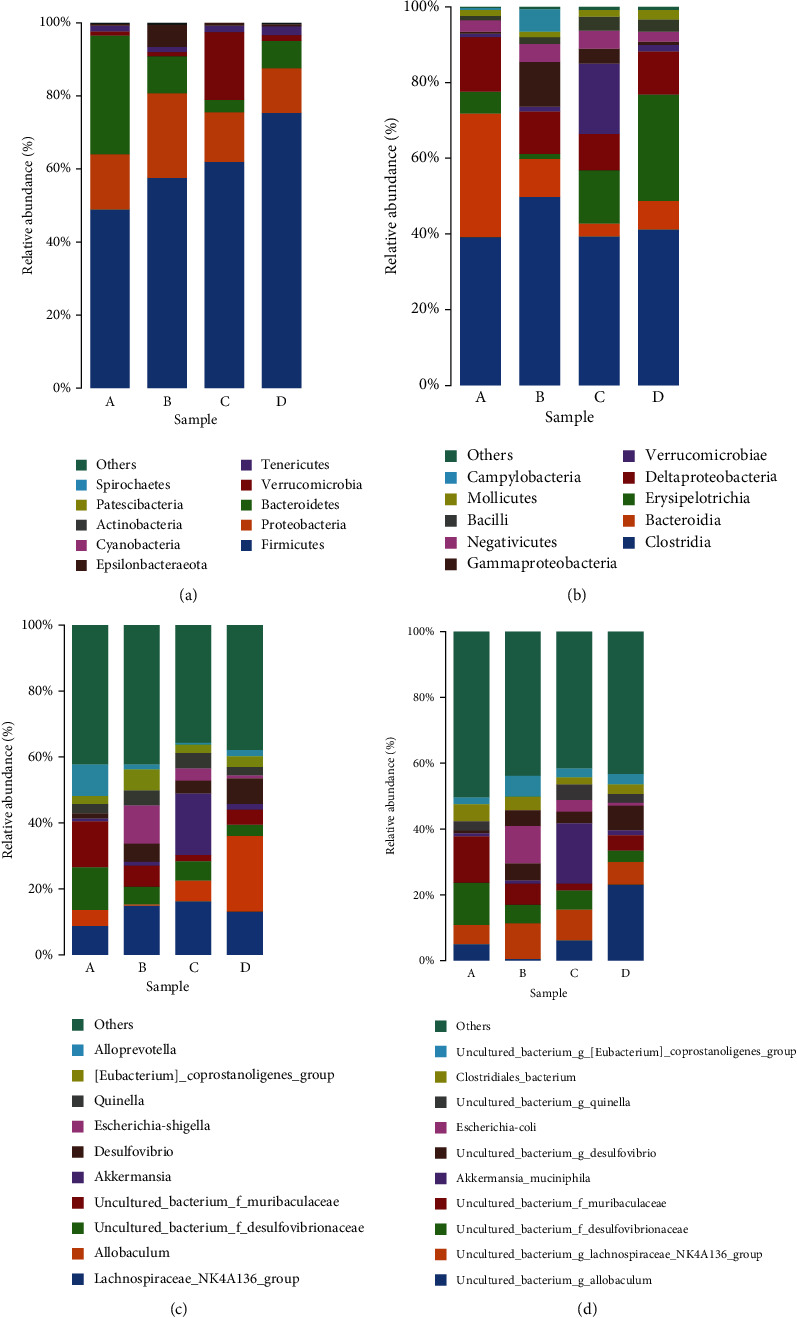
Taxonomic analysis results showing the relative abundances of (a) microbiota at phylum level, (b) microbiota at class level, (c) microbiota at the genus level, and (d) intestinal microbiota at the species level.

Taxonomic analyses were also carried out to compare the relative abundance of the flora at the phylum, class, genus, and species level. At the phylum level, the main phyla are *Firmicutes*, *Proteo-bacteria*, *Bacteroidetes*, *Verrucomicrobia*, and *Tenericutes* which account for over 95% of the total. The results (Figure 12(a)) show that the relative abundance of *Proteobacteria* is increased in the LPS group but the addition of TL3 restores it back to its original value. Interestingly, the addition of TL3 can be seen to significantly increase the abundance of *Verrucomicrobia* (*p* < 0.01) whereas the levels of the other bacterial phyla are not significantly different.

The results obtained at class level are similar to those observed at phylum level. That is, TL3 reverses the LPS-induced increase in relative abundance of *Gammaproteobacteria* (Figure 12(b)), and it also reverses the significant decrease in the relative abundance of *Verrucomicrobiae* (*p* < 0.01).

The results of the genus level analysis (Figure 12(c)) show that there is an increase in the relative abundance of *Escherichia-Shigella* in the LPS group and a decrease in the relative abundance of *Akker-mansia* and *Allobaculum*. Once again, these trends are reversed by the addition of TL3.

In order to further show the differences between the samples, a species level analysis was performed. The results (Figure 12(d)) were found to be consistent with those obtained at genus level. The relative abundance of *Escherichia coli* is increased in the LPS group, and the relative abundances of *Akkermansia muciniphila* and *uncultured bacterium g. Allobaculum* are decreased. As can be seen, the addition of TL3 reversed these trends.

### 3.9. TL3 Regulates the Key Microorganisms Induced by LPS in the Guts of Rats

**Figure 13:**
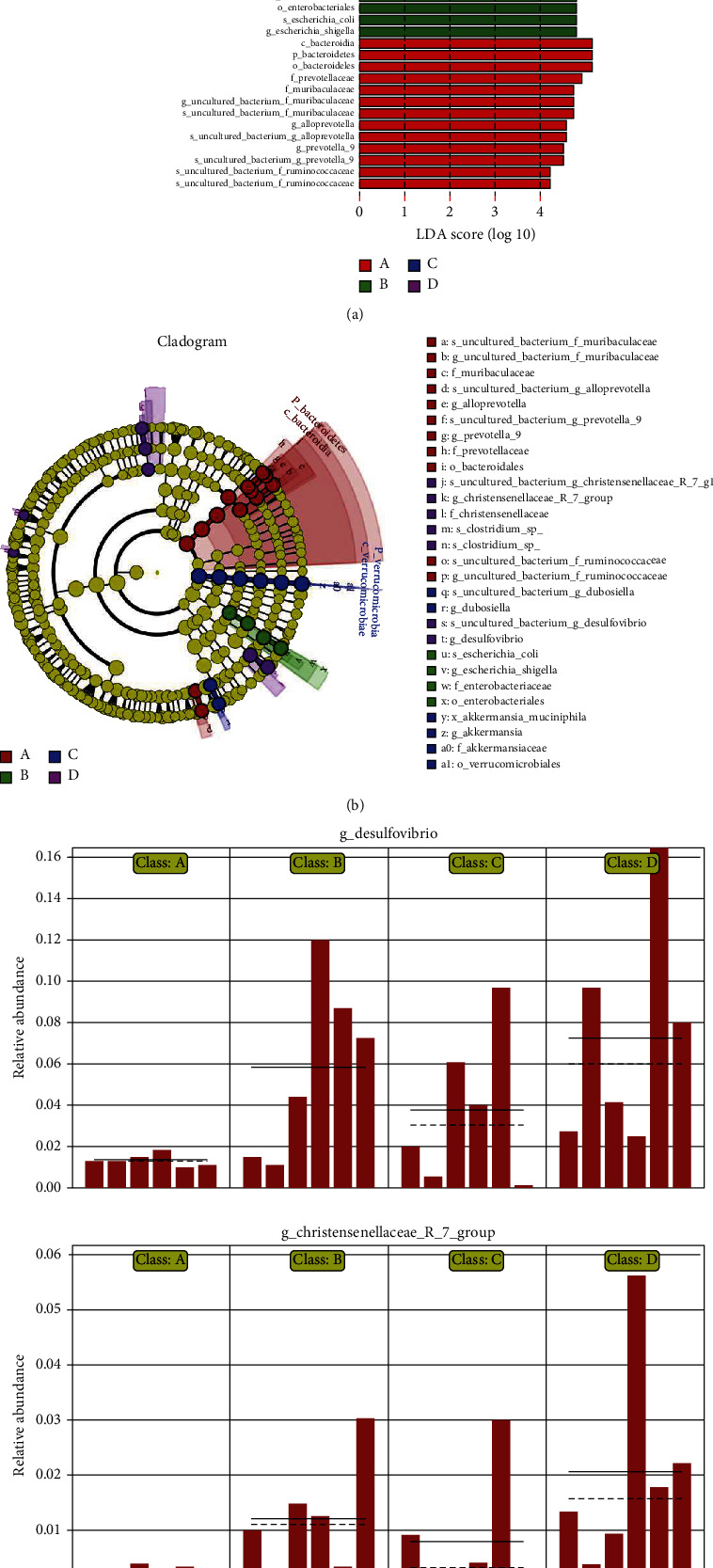
LDA results showing (a) a histogram of the LDA values obtained, (b) the corresponding cladogram obtained, and (c) a comparison of the LEfSe abundances between the different experimental groups.

Linear discriminant analysis (LDA) was used to determine the number of distinct bacterial clades with LDA ≥ 4.0. The results, shown in Figures 13(a) and 13(b), indicate that 32 distinct clades were found at all taxonomic levels. The LDA effect size (LEfSe) method of analysis was further used to identify the high-dimensional biomarkers in each group of gut microbiota, giving the results shown in Figure 13(c).

The results show that the addition of TL3 greatly increases the abundance of *Akkermansia muciniphila*, a probiotic that helps reduce the inflammation caused by LPS. It also effectively reduces the relative abundances of harmful bacteria induced in rats by LPS, including *Escherichia coli*, *Escherichia Shigella*, and other *Enterobacteriaceae*.

## 4. Discussion

### 4.1. Protective Effect of *B. coagulans* TL3 on Intestinal Pathological Injury Induced by LPS

Goblet cells are generally distributed between the columnar epithelium of small intestinal mucosa and are a kind of unicellular glands. When goblet cells mature, the cytoplasm is full of mucinogen granules, which can secrete a large amount of mucin, and the end is acidic containing sialic acid and sulfated lipids, thus forming a mucosal barrier to protect intestinal tissue [42–44]. Therefore, we use H&E staining and Alcian blue staining to show the cecal tissue structure and the number of goblet cells and then reflect the functional state of the rat cecum. Jayaraman et al.'s research results showed that the addition of Bacillus significantly increased the ratio of villi length to crypt depth, leaving the intestines intact without obvious pathological changes and effectively inhibiting the intestinal damage caused by *Clostridium perfringens* [45]. Wu et al.'s research results showed that the addition of *Bacillus coagulans* significantly increased the ratio of intestinal appearance height to crypt depth and the number of goblet cells, protecting the intestine from damage caused by *Clostridium perfringens* [46]. Similar results in this study, the number of goblet cells in the LPS group was significantly less than that in the CON group, and the structure of the cecum was disordered. Compared with LPS group, the number of goblet cells in HBC group and LBC group increased significantly, and there was no difference between HBC group and LBC group and CON group. These results suggest that TL3 can effectively improve the cecal injury induced by LPS in mice. These results suggest that TL3 can effectively improve the cecal injury induced by LPS in mice.

### 4.2. Protective Effect of *B. coagulans* TL3 on Oxidative Damage Induced by LPS

Under normal conditions, the production and removal of ROS in the body exists in a state of dynamic equilibrium. However, if free radicals accumulate in cells and tissues and exceed the removal ability of antioxidants, the balance will be broken, causing the body to undergo oxidation. When subjected to such stress, homeostasis is destroyed, and oxidative damage occurs to cells and tissues [7]. SOD is an important enzyme that scavenges oxygen free radicals and can prevent the peroxide reaction [47]. GSH-Px can remove harmful metabolites in cells and protect the structural and functional integrity of cell membranes [48]. MDA is the end product of lipid oxidation, and so, its concentration indirectly reflects the degree of damage caused to the membrane system [49]. CAT is one of the key enzyme proteins that removes peroxidation factors and can thus prevent damage occurring to the body after oxidation or phagocytosis [50]. Related research results show that *Bacillus coagulans* can relieve the intestinal oxidative stress of fish exposed to cadmium by restoring the activities of GSH-PX, SOD, and CAT and reducing MDA [51]. Consistent with the results, in this experiment, SOD, GSH-PX, and CAT in the LPS group were significantly reduced, and MDA was significantly increased, indicating that LPS caused oxidative stress in mice. Adding the *Bacillus coagulans* group significantly improved these changes, indicating that *Bacillus coagulans* can effectively protect the intestinal tract from damage caused by LPS.

Nuclear factor E2-related factor 2 (Nrf2) is the main regulator of antioxidant substances in cells and thus plays an important role in body's antioxidant regulatory system [52]. Under oxidative stress conditions, Kelch-like ECH-associated protein 1 (Keap1) undergoes a redox reaction as it contains terminal cysteine residues. This causes the Keap1 to become dissociated from Nrf2. The Nrf2 can then bind to the coactivator cAMP response element-binding (CREB) protein and then undergo dimerization with Maf to form Nrf2–Maf dimers. The Nrf2 can then bind to the antioxidant responsive element (ARE) to induce downstream antioxidant gene expression [53, 54]. The downstream proteins of NRF2, NADH quinone oxidoreductase 1 (NQO1), and heme oxygenase 1 (HO-1) are important antioxidant-related enzymes that can provide protection against various forms of stress and also play a vital role in the Nrf2 signaling pathway [55, 56]. Relevant studies have shown that honeysuckle polyphenols can inhibit oxidative stress in rats fed a high-fat diet by activating the Nrf2/NQO1/HO-1 pathway. The results of Bai et al.'s research show that Bacillus subtilis fmbJ improves the expression of Nrf2, HO-1, SOD, and GPx, so that broiler chickens have antioxidant capacity [57]. Similarly, the results of Wang et al.'s study showed that the regulation of the Nrf2/Keap1 pathway by *Bacillus amyloliquefaciens* SC06 is involved in alleviating the oxidative stress induced by H2O2 in porcine epithelial cells [58]. Consistent with the above results, the results of this study show that LPS can significantly increase the expression of Nrf2, NQO1, HO-1, GPX, and GCLC in the cecum of rats, while *Bacillus coagulans* TL3 significantly inhibits the changes of these indicators, indicating that *Bacillus coagulans* TL3 can protect the intestine from oxidative stress damage through the Nrf2 signaling pathway.

### 4.3. Protective Effect of *B. coagulans* TL3 on Inflammatory Injury Induced by LPS

Cytokines can be divided into two types: those that are proinflammatory (e.g., TNF-*α*, IL-6, and IL-1*β*) and those that are anti-inflammatory (e.g., IL-4) [59]. It has been shown that *Lactobacillus plantarum* CECT7315/7316 can reduce the levels of IL-1*β* and IL-6 induced in rats by LPS and also increase the level of IL-10 *Bacillus coagulans*, thus reducing inflammation [60]. *Lactobacillus reuteri* CRL1101 has also been shown to downregulate the levels of cytokines IL-1*β*, IL-6, and TNF-*α* induced in the sera of mice by LPS [61]. The present study indicates that probiotics can significantly improve the enzymatic indicators in rat serum. The serum levels of IL-6, IL-1*β*, and TNF-*α* in the rats in our experimental LPS group were significantly increased compared with the control group, and the IL-4 content was decreased. However, the addition of TL3 significantly reversed these trends. Our results show that *B. coagulans* can improve the immune function of the body by inhibiting the secretion of proinflammatory cytokines and increasing the secretion of anti-inflammatory cytokines.

Studies have shown that LPSs are components of the cell walls of Gram-negative bacteria. LPS is released when such bacteria die. It can then bind to the toll-like receptor 4 (TLR4) on the surfaces of cells to activate the nuclear transcription factor *κ*B (NF-*κ*B) signaling pathway [ 41]. TLR4 is a type I transmembrane protein which is expressed on cell membranes. There is evidence that TLR4 plays an important role in the occurrence and acceleration of inflammation [62, 63].

The NF-*κ*B signaling pathway is located downstream of the TLR4/MYD88-dependent signal transduction process. Under normal circumstances, the combination of NF-*κ*B and I*κ*B is in a static state. After external stimulation, the TLR4/MYD88 signaling pathway is activated, and the I*κ*B kinase complex is stimulated. This causes the I*κ*B to be activated, which activates previously degraded NF-*κ*B [64–66], thereby regulating the expression of target genes such as IL-1*β*, IL-6, and TNF-*α* [67]. Relevant studies have shown that Bacillus SC06 can significantly downregulate the gene expression of TNF-*α* and IL-1*α* in the intestinal mucosa, but upregulate the transcription of IL-6 and IL-8, thereby improving the structure of the intestinal mucosa to increase the intestinal epithelial cell barrier and immune function [68]. The results presented in this study show that LPS significantly upregulates the expression of inflammatory pathway proteins TLR4, MYD88, NF-*κ*B, IL-1*β*, IL-6, and TNF-*α*. Moreover, the addition of the strain TL3 significantly reverses this behavior. This shows that TL3 can inhibit the activation of NF-*κ*B and expression of downstream proinflammatory factors and thus protect the ceca of rats from LPS-induced inflammatory effects.

### 4.4. Protective Effect of *B. coagulans* TL3 on Mechanical Barrier Damage Induced by LPS

The intestinal mucosa is a mechanical barrier that is composed of intestinal mucosal epithelial cells, intercellular TJs, and bacterial membranes. The intestinal mucosa can become damaged due to inflammation or stress response. A damaged mucosa can allow a large number of inflammatory factors to enter the portal vein. In severe cases, the lymphatic system can be affected causing systemic inflammation [69].

TJs are comprised mainly of occludin, claudins, and zonula occludens (ZO) [70]. The structure of the TJs in the intestine is highly dynamic and the degree of sealing changes when the body is under pathological conditions or is subjected to external stimuli [71]. Our results show that the expression levels of the TJ-related proteins claudin-1, occludin, and ZO-1 were significantly reduced in the rats in the LPS group. Moreover, the addition of TL3 had a good inhibitory effect on these LPS-induced changes. That is, the addition of the probiotic (HBC and LBC groups) significantly increased the expression levels of claudin-1, occludin, and ZO-1, compared to the LPS group. It thus reduced intestinal permeability by protecting the integrity of the TJs and maintaining intestinal barrier functions.

### 4.5. *B. coagulans* TL3 Regulates Intestinal Flora to Protect Intestinal Injury Caused by LPS

The floras in the human intestine are large in number and diversity and include probiotics and symbiotic and pathogenic bacteria that form a complex microecosystem. If the balance that exists between the intestinal flora is disrupted, it can affect the mucous membranes. There may be excessive consumption of layers and accelerated apoptosis of intestinal mucosal epithelial cells, resulting in damage to the intestinal mucosal barrier [72]. Moreover, the invasion of a large number of pathogenic bacteria will trigger a strong intestinal immune response. This will lead to an increase in the secretion of various inflammatory factors in the intestine and will ultimately result in digestion and absorption dysfunction [73].

At the phylum level, we found that there was an increased abundance of *Proteobacteria* in the intestinal flora of the rats in the LPS group. Their abundance in the rats in the HBC group, however, was essentially the same as that in the control group. *Proteobacteria* are Gram-negative bacteria. An increase in *Proteobacteria* concentration indicates an increase in the number of pathogenic bacteria that can produce endotoxins. This can lead to an increase in the concentration of LPS in the serum and cause endotoxemia. Studies have shown that the integrity of the intestinal mucosal barrier is related to the abundance of *Proteobacteria*. That is, an increase in *Proteobacteria* abundance can destroy the original intestinal mucosal barrier, increasing the permeability of the intestine and triggering intestinal inflammation [59].

Our LEfSe abundance comparison charts (Figure 13(c)) show that the TL3 strain significantly increases the relative abundance of the beneficial bacterium *Akkermansia muciniphila* (belonging to the phylum Verrucomicrobia), which can degrade intestinal mucin to produce short-chain fatty acids. It is negatively related to obesity, diabetes, cardiometabolic diseases, and low-grade inflammation [74]. Studies have also shown that it can adhere to intestinal epithelial cells and enhance the integrity of intestinal epithelial monolayer cells in vitro and enhance the barrier function of damaged intestines [75].

We also found that the addition of TL3 can significantly reduce the relative abundance of harmful bacteria *Escherichia coli*, *Escherichia-Shigella*, and other *Enterobacteriaceae*. Studies have found that certain conditional pathogens or LPS-producing bacteria, such as *Enterobacteriaceae* that are characterized by endotoxin production, are closely related to metabolic endotoxin levels and systemic inflammation [76]. Our results show that LPS induces significant changes in the intestinal flora of the rat specimens. After TL3 is added, the types and numbers of anti-inflammatory and antioxidant microbial flora are increased, and the harmful bacteria that induce inflammation and oxidative stress in the intestinal flora are reduced. This indicates that TL3 can alleviate LPS-induced cecal damage in rats by regulating their intestinal flora.

*B. coagulans* is a spore-producing lactic acid bacteria, which has the ability to improve feed conversion rate and intestinal flora balance and ultimately improve the growth performance of animals and has better storage characteristics than traditional lactic acid bacteria probiotics. It can also regulate immune function, help the immune system prevent infection, and minimize inflammation-related tissue damage. *Bacillus coagulans* can play a beneficial role as a feed additive. The mechanism of action of *Bacillus coagulans* in animal husbandry is worthy of further study.

## 5. Conclusions

In conclusion, *Bacillus coagulans TL3* inhibits LPS-induced inflammatory effects and oxidative stress in rat cecal tissue by regulating the TLR4/MyD88/NF-*κ*B and Nrf2 signal pathways and modulating intestinal microflora.

## Data Availability

Data is contained within the article.
